# Multicentric Breast Abscesses in a Patient Who Had COVID-19

**DOI:** 10.31486/toj.21.0095

**Published:** 2021

**Authors:** Mary Van Wert, Michael Ghio, Caroline Graham, Dana Smetherman, Ronda Sanders, Ralph Corsetti

**Affiliations:** ^1^ Department of Surgery, Ochsner Clinic Foundation, New Orleans, LA; ^2^ Department of Surgical Oncology, Lakeview Regional Medical Center: A Campus of Tulane Medical Center, Covington, LA; ^3^ Department of Radiology, Ochsner Clinic Foundation, New Orleans, LA; ^4^ The University of Queensland Faculty of Medicine, Ochsner Clinical School, New Orleans, LA; ^5^ Department of Pathology, Ochsner Clinic Foundation, New Orleans, LA

**Keywords:** *Abscess*, *breast*, *COVID-19*, *vasculitis*

## Abstract

**Background:** Coronavirus disease 2019 (COVID-19) is recognized as a multisystem disease affecting the whole body, with new complications from the disease being described on an almost-daily basis.

**Case Report:** We report the case of a 50-year-old female with a medical history of diabetes and silicone breast implants who developed right-sided, multicentric breast masses after a prolonged hospitalization for COVID-19 infection complicated by renal failure requiring dialysis. The patient noted an onset of breast pain and masses, and subsequent imaging demonstrated multiple similar oval masses. She underwent biopsy and operative debridement of the lesions and recovered appropriately. Results were consistent with sterile abscesses that were considered secondary to a vasculitis-like process associated with COVID-19 infection.

**Conclusion:** To our knowledge, this case is the first account of breast pathology associated with a diagnosis of COVID-19 in the medical literature and encourages systematic evaluations of patients with coronavirus infections, including breast examinations.

## INTRODUCTION

Coronavirus disease 2019 (COVID-19) is associated with numerous systemic manifestations, including various vasculitis and vasculitis-like processes, coagulopathies, and other conditions.^1,2^ As demonstrated in the lung, abscess formation can complicate COVID-19.^3^ To our knowledge, breast involvement has not previously been reported as a systemic effect of COVID-19 infection. We present a case of multicentric breast abscesses complicating COVID-19 infection.

## CASE REPORT

A 50-year-old female presented to the clinic in August 2020 with a complaint of multiple palpable nodules of the right breast. The patient had a complex medical history, including hypertension, hyperlipidemia, diabetes, and tetralogy of Fallot with a bioprosthetic valve. She had had bilateral subpectoral silicone breast implants in 2013, with no postoperative complications. The patient had no personal history or family history of breast pathology.

In spring 2020, she experienced a 68-day hospitalization for respiratory failure secondary to COVID-19 infection. During this initial COVID-19 hospitalization, the patient required paralytics for ventilation, continuous renal replacement therapy for acute kidney failure, broad spectrum antibiotics for staphylococcal bacteremia and ventilator-associated pneumonia, and treatment of an upper gastrointestinal bleed. The patient was never proned. She was transferred to a long-term acute rehabilitation facility and eventually was discharged to home in good condition in late May 2020.

**Figure 1.**
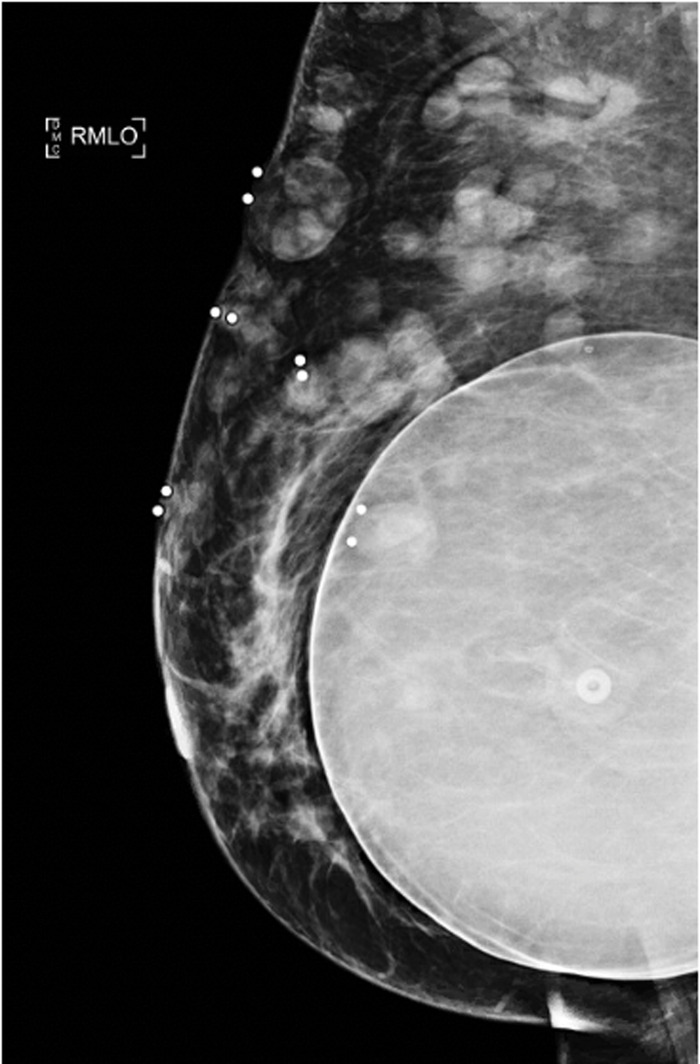
Right mediolateral oblique (RMLO) mammogram demonstrates breast masses.

**Figure 2.**
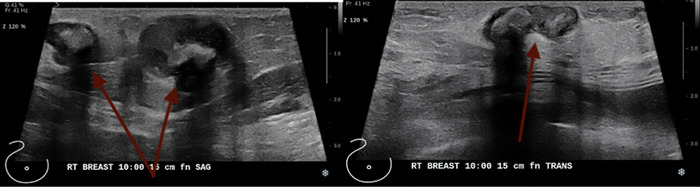
Right breast ultrasound demonstrates multiple abscesses at the 10 o’clock position 15 cm from the nipple.

**Figure 3.**
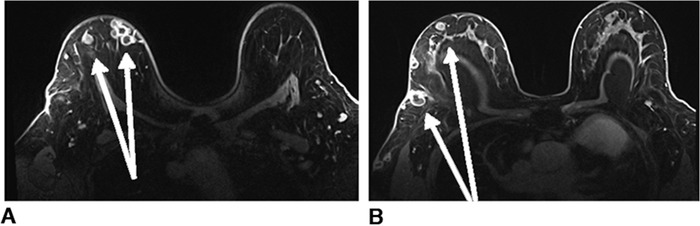
Bilateral breast magnetic resonance imaging shows (A) multiple breast abscesses (arrows) in the right breast and (B) a normal (arrows) left breast.

When the patient presented in August 2020, the nodules were tender to palpation without drainage, warmth, or erythema. Right breast mammography (Figure 1) and ultrasound (Figure 2) demonstrated multiple oval masses in the right breast extending into the axilla and the upper arm. On mammography, the masses were of equal density with well-circumscribed margins. On ultrasound, the masses were oval and parallel to the skin surface, with a heterogeneous internal echotexture. A mildly enlarged right axillary node with mild focal cortical thickening (4 mm) was also noted. The patient underwent bilateral magnetic resonance imaging (MRI) (Figure 3) for further characterization of the lesions. On breast MRI, the clinical findings in the right breast corresponded to multiple rim-enhancing masses with intrinsic T1 and T2 hyperintensity. Mild right axillary adenopathy was again noted. The findings were favored to be infectious in etiology—fungal, mycobacterial, or viral—with likely reactive right axillary lymphadenopathy.

**Figure 4.**
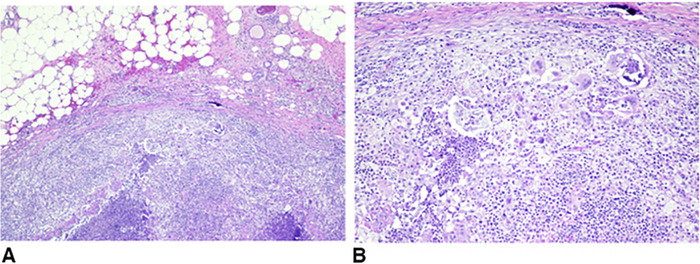
Pathology images from the right breast excision. (A) Fat necrosis and granulomas. (B) Histiocytes, lymphocytes, neutrophils, and giant cells.

The patient desired excision of some of the most symptomatic lesions. She was taken for surgical excisional biopsy, and a cluster of nodules on the right lateral breast was removed. Grossly, the nodules had the appearance of caseating granulomas with liquid cores. The creamy exudate was not malodorous. Cultures were sterile for bacterial, viral, and fungal sources. Pathology (Figure 4) showed multiple rubbery, soft, lobulated, yellow hemorrhagic tissue fragments. The cut sections demonstrated multiple light yellow areas resembling fat necrosis. The final pathologic diagnosis was benign fibroadipose breast tissue with consolidated abscess formation and associated fat necrosis and microcalcifications. The sample was negative for atypia or malignancy.

The patient returned to clinic 1 month postoperatively, pleased with her results and with well-healing scars without any additional concerns for reformation of the abscesses.

## DISCUSSION

Published evidence in the COVID-19 era demonstrates both microvascular and macrovascular thrombosis involving vessels in multiple organs.^2^ Vessel thrombosis can result from endothelial cell damage with subsequent dysfunction and oftentimes apoptosis. COVID-19 can be associated with a hypercoagulable process reflected by elevated levels of factor VIII, von Willebrand factor, and fibrinogen 1. A number of published cases report COVID-19–related thrombotic events involving the central nervous system, renal system, pulmonary system, aorta and major arterial vascular system, and skin lesions in the lower and upper extremities.^4-16^ The majority of reports on this subject involve youth and adolescents. A concurrent but separate process can also occur in patients with COVID-19 related to inflammatory lymphocytic vasculitis or other vasculitis-like process.^2,16^ Suggested mechanisms include increased complement activation or disseminated intravascular coagulation.^16^ Iba et al discuss a 30% increase of deep venous thrombotic events that are secondary to these vasculitis processes in patients with COVID-19.^1^ These processes can result in necrotic lesions that Galván Casas et al observed in approximately 6% of patients with skin lesions.^15^ Galván Casas et al also reported that the incidence of necrotic changes corresponded to increasing age and severity of COVID-19 and that necrosis may be associated with an increased mortality risk by up to 10%.^15^

In this case report, we describe one of the first examples, to our knowledge, of a breast pathology likely secondary to COVID-19. We suggest that the observed abscess formation was associated with a similar thrombotic process as described in the literature, likely a vasculitis-like process. The areas of necrosis are consistent with reported findings in other systems, particularly in cutaneous skin reports and cardiac cells undergoing necrosis.^15,17^ No available research provides a definitive explanation regarding the abscess formation. While our patient's course would not likely have been altered by the knowledge that the abscesses were not infected, clinicians should consider sterile abscesses as a possibility in patients presenting with breast masses following a recent COVID-19 infection. An awareness of previous abscesses following COVID-19 may allow surgeons to confidently delay surgery and its associated risks and instead monitor breast masses in patients who have no other indications of malignancy on examination or imaging.

Research is needed to elucidate the exact mechanism in the development of these sterile breast abscesses. Conclusions are limited by the fact that ours is the only case reported in the literature. In addition, follow-up with this patient has been short, with only one visit one month postoperatively. Nevertheless, recognizing the possibility of breast involvement in patients after prolonged hospitalization for COVID-19 is important.

## CONCLUSION

In this case, we describe one of the few known breast complications related to COVID-19 infection, specifically, the development of sterile abscesses. We report this case to highlight the associated imaging and pathologic findings. Because of the uncertainty of the etiology of these lesions and concurrent symptoms, the patient desired elective surgical excision for both diagnostic and therapeutic reasons. However, avoiding such interventions in patients with a similar imaging or biopsy pattern may be possible.

## Notes

*This article meets the Accreditation Council for Graduate Medical Education and the American Board of Medical Specialties Maintenance of Certification competencies for Patient Care and Medical Knowledge.*
